# Stress, breast cancer, and the epigenetic echo: moving beyond the polygenic narrative?

**DOI:** 10.3389/fonc.2026.1865168

**Published:** 2026-06-02

**Authors:** Shiqiong Zhou, Qinghua Ke

**Affiliations:** Department of Chemoradiotherapy, The First People’s Hospital of Jingzhou, Jingzhou, Hubei, China

**Keywords:** breast cancer, clinical guidance, competing risks, DNA methylation, epigenetics, polygenic risk score, risk modification, stressful life events

## Introduction

The prospective study by Azzi and colleagues, with its 36−year follow−up of over 10 000 women from the Finnish Twin Cohort, reports that cumulative stressful life events (SLEs) are associated with a modest but significant increase in breast cancer (BC) risk (HR 1.04 per event, 95% CI 1.00–1.07) (1). The study’s twin−pair design and integration of DNA methylation (DNAm) data are notable strengths, suggesting that the SLE-BC association is unlikely to be explained by shared genetic predisposition. The long follow-up, twin-pair discordance analysis, and replication in cancer-free pairs lend weight to these findings. Nevertheless, this commentary argues that the study falls short of establishing causation, and several critical questions remain—questions that have direct implications for clinical risk assessment and prevention. We provide a critical appraisal of these limitations, focusing on the weak polygenic signal, the non-causal nature of the epigenetic findings, the absence of intervention data, and underexplored birth cohort effects, and —most importantly for the clinician—what can and cannot be recommended to patients today. We then outline a more responsible path forward for clinical translation.

## The weak polygenic signal: competing risks as one likely explanation

The association between the polygenic risk score for breast cancer (PRS−BC) and incident BC (HR 1.03 per SD) in this cohort is notably weaker than in larger European studies, where per−SD effect sizes are typically around 1.28–1.35 (e.g., Mars et al. (2) reported HR 1.55 for highest vs. lowest decile). The authors attribute this to longer follow−up and environmental risk masking. An alternative, and underexplored, explanation is differential survival or competing mortality. Given that the cohort was born before 1958, selective attrition due to cardiovascular disease and suicide—both potentially linked to chronic stress—could have attenuated the observed PRS−BC effect. Competing risks are not the only possible explanation, but they are a strong candidate. To formally test this, future studies should apply a Fine−Gray subdistribution hazard model, treating cardiovascular mortality and suicide as competing events. Such an analysis would clarify whether the apparent attenuation is driven by competing risks rather than by biological masking alone.

## DNA methylation findings: correlation, not causation

The DNAm findings are intriguing, and the within−MZ−pair replication strengthens their credibility (3). However, the study stops short of establishing mediation, a point the authors acknowledge due to limited statistical power. While the 42 CpG sites associated with both SLE exposure and BC risk are hypothesis-generating, we should avoid overinterpreting them as causal. DNAm was measured in blood at a single time point decades after the reported SLEs, raising the possibility that these peripheral marks reflect a chronic stress phenotype rather than a causal breast tissue pathway. Rather than calling for unfeasible serial breast biopsies, we propose more realistic designs. These include using surrogate tissues such as buccal swabs or breast ductal lavage, and leveraging ongoing efforts like the Human Tumor Atlas Network. Additionally, blood DNAm remains valuable: several PACE consortium studies have shown cross-tissue correlations for stress-associated CpG sites, allowing peripheral methylation to serve as a reasonable proxy when interpreted with caution.

## Biological mechanisms: from association to plausibility

A major criticism of the original study is the lack of mechanistic data linking psychological stress directly to breast carcinogenesis. While the authors of this commentary agree that causation is not established, it is important to acknowledge that the biological plausibility has been strengthened by recent experimental work. Chronic stress activates the hypothalamic−pituitary−adrenal (HPA) axis and the sympathetic nervous system, leading to elevated glucocorticoids and catecholamines. In animal models and human breast cancer cell lines, these stress mediators have been shown to: (i) induce DNA damage via oxidative stress and downregulation of DNA repair enzymes (e.g., OGG1, BRCA1); (ii) promote epithelial−mesenchymal transition and cancer stem cell phenotypes; (iii) impair anti−tumor immune responses by suppressing NK cells and cytotoxic T lymphocytes; and (iv) alter the tumor microenvironment through increased angiogenesis and matrix metalloproteinase activity (4–6). However, most of these data come from rodent studies or *in vitro* systems, and direct evidence in human breast tissue—especially in non−cancerous breast epithelium of stressed women—remains sparse. Thus, while biological plausibility exists, it does not substitute for causal proof. Future prospective studies should collect serial biospecimens (blood, buccal cells, and where feasible, breast ductal fluid) to examine whether stress−induced molecular changes precede and predict BC development.

## Can stress−reducing interventions modify breast cancer risk? A missing clinical link

The study raises an unaddressed but clinically crucial question: do stress-reducing interventions modify BC risk? The authors implicitly suggest that SLEs are an independent risk factor, yet no data on coping strategies, social support, or subsequent stress exposure were collected. Before proceeding, it is useful to define what we mean by “direct carcinogen.” Here we use the term to mean an exposure that directly induces DNA damage or drives clonal expansion in breast epithelium through a well-established, dose-dependent mechanism (e.g., ionizing radiation or tobacco carcinogens). Stress is unlikely to meet this definition. Instead, it should be viewed as a risk modifier—a factor that operates through behavioral, neuroendocrine, or immune pathways to influence the effects of other exposures.

Randomized trials of long-term stress management for cancer prevention are neither feasible nor ethical. However, observational and quasi−experimental evidence is not entirely absent. For example, a large Swedish registry study found that women prescribed selective serotonin reuptake inhibitors (SSRIs) for prolonged stress or mild depression had no reduction in BC risk, but those who participated in structured stress reduction programmes (e.g., mindfulness−based stress reduction) showed improved adherence to mammography screening—though BC incidence itself was not significantly altered. More compellingly, a Finnish population−based study noted that women with high workplace stress who were exposed to an employer−mandated mental health support program had a modestly lower all−cause mortality, but BC−specific analyses were underpowered. These examples illustrate the gap: we have hints, but no robust evidence that stress reduction lowers BC risk.

To move forward, we propose the following pragmatic designs, which go beyond theoretical suggestions:

 Registry−based medication studies: Linking prescriptions of stress−related medications (e.g., SSRIs, benzodiazepines) to BC incidence, while carefully controlling for indication bias using negative control outcomes (e.g., injuries). Confounding by indication is a major concern, as anxiety or depression itself may be associated with BC risk through shared biological or behavioral pathways.
 Target trial emulation using electronic health records: Comparing BC risk in women exposed versus unexposed to policy changes affecting work−related stress or bereavement leave. Key elements would include defining eligibility as women with no prior cancer, exposure as a major policy change (e.g., extended bereavement leave or implementation of a national stress−leave insurance), follow−up from policy implementation to BC diagnosis or censoring, and outcomes as incident BC. Such emulations can provide the strongest quasi−experimental evidence short of a randomized trial.
 Mendelian randomization: Using genetic variants for stress reactivity or cortisol regulation as instrumental variables. Strong instruments (F−statistic >10) and sensitivity analyses for pleiotropy (e.g., MR−Egger, weighted median) are essential before any causal interpretation can be attempted.

## Birth cohort effects: gene–environment interplay or hormonal shifts?

The observed birth cohort effect—higher HR for SLEs in women born before 1950 versus after—is fascinating but underexplored. The authors speculate about historical trauma (7), but an alternative biological explanation involves changing reproductive patterns. In Finland, secular trends show a decline in mean age at menarche from approximately 14.5 years in the 1920s to 12.7 years in the 1960s, and a decrease in parity from 2.5 to 1.6 children per woman over the 20th century. These changes, together with increased use of oral contraceptives and hormone replacement therapy, have led to higher cumulative estrogen exposure in later−born cohorts. Such hormonal shifts could modify stress reactivity via the HPA axis (8). Future studies should formally test interactions between SLEs, reproductive history, and lifetime estrogen exposure using historical cohort data or pooled analyses with harmonized reproductive variables.

## Clinical guidance: what to tell patients today?

The most immediate clinical implication of the Azzi study is not to add SLEs to risk calculators, but to recognize that patients with high stress burden may warrant closer attention to adherence with established screening guidelines. As a practicing oncologist and journal editor, I often encounter the question: “Doctor, does stress cause breast cancer, and what should I do about it?” Based on current evidence, here is a responsible answer:

- Do not change medical follow−up intervals or pharmacological prevention thresholds based on stress alone. For example, in a patient with high stress burden but otherwise average risk, we would not recommend earlier screening (e.g., starting mammography at age 35) solely on the basis of stress. There is no evidence that earlier or more frequent screening improves outcomes in this subgroup.
- Do use the presence of high stress burden as a prompt to ensure that patients are not missing routine mammograms. Stressed individuals often skip preventive appointments. A brief discussion during annual visits can improve adherence.
- Do discuss lifestyle factors (alcohol reduction, physical activity, healthy weight) that both lower BC risk and improve stress resilience.
- Do offer or refer to evidence−based stress reduction programmes (e.g., cognitive−behavioral therapy, mindfulness, exercise interventions)—not because these programmes have been proven to lower breast cancer risk, but because they improve quality of life, mental health, and cardiovascular outcomes. Should future trials demonstrate a BC risk reduction, that would be a welcome bonus, but it should not be the primary justification.
- Do consider a simple clinical risk assessment: For a stressed patient with additional risk factors (e.g., family history, known germline mutation, dense breasts), the stress factor does not alter the threshold for genetic counselling or risk-reducing medication. Those decisions remain based on established models (Tyrer−Cuzick, Gail, etc.).

In short, stress should inform patient counselling about healthy behaviors and screening adherence—but not change clinical thresholds for risk prediction or early detection.

## Discussion and future directions: from association to clinical action

Azzi and colleagues have shown that SLEs are associated with BC risk independent of common genetic variants. However, the transition from association to causation—and from epigenetics to clinical action—remains large. To responsibly advance this field, we propose four specific research priorities, each with a concrete design recommendation:

1. Intervention pragmatics: Do stress−reducing interventions measurably alter breast cancer incidence in target trial emulations or registry−based analyses?

Design: Emulate a trial where the intervention is “access to an employer−mandated stress reduction program” and the outcome is BC incidence over 10 years, using difference−in−differences analysis.

2. Peripheral epigenetics: Are there validated peripheral epigenetic biomarkers that can serve as proxies for breast tissue stress responses?

Design: Simultaneous collection of blood, buccal cells, and breast ductal fluid (from high−risk women undergoing prophylactic mastectomy or diagnostic lavage) to identify CpG sites that correlate across tissues.

3. Reproductive modification: How do reproductive and hormonal factors modify the SLE−BC association?

Design: Pooled analysis of three Nordic cohorts with harmonized variables for age at menarche, parity, oral contraceptive use, and hormone replacement therapy, testing interaction terms.

4. Competing mortality: What is the impact of competing mortality (cardiovascular disease, suicide) on the observed PRS−BC attenuation in older cohorts?

Design: Fine−Gray subdistribution models with cause−specific hazards, applied to the Finnish Twin Cohort or similar long−run datasets.

In addition to the cited literature, recent mechanistic work has strengthened the biological plausibility of stress−attributed BC incidence and progression (4, 5), and emerging evidence points to chronic−stress epigenomic markers in BC phenotypes (6). However, these findings do not yet overcome the causal and methodological limitations discussed here.

## Conclusion

For the clinician and the epidemiologist, the message from Azzi and colleagues is important but incomplete. Stressful life events are associated with modestly increased breast cancer risk independent of common genetics, and the epigenetic signals are hypothesis−generating. However, the weak polygenic signal may reflect competing mortality, the DNA methylation findings fall short of causality, the role of stress reduction remains untested, and birth cohort effects suggest hormonal interactions that require further study. Biologically, there are plausible pathways linking chronic stress to breast carcinogenesis, but direct human evidence is lacking. Clinically, we can responsibly advise patients that while stress is not a proven direct cause, managing stress improves overall health and may help maintain adherence to cancer prevention behaviors. The path forward lies in pragmatic intervention designs, careful handling of competing risks, and surrogate tissue epigenetics. The evidence is not yet sufficient for formal integration of stress into breast cancer prediction models. For now, stress should inform patient counselling about adherence to screening and healthy lifestyles—but not change clinical thresholds for risk prediction or early detection.
